# Improved Prediction of miRNA-Disease Associations Based on Matrix Completion with Network Regularization

**DOI:** 10.3390/cells9040881

**Published:** 2020-04-03

**Authors:** Jihwan Ha, Chihyun Park, Chanyoung Park, Sanghyun Park

**Affiliations:** 1 Department of Computer Science, Yonsei University, Seoul 03722, Korea; jihwanha@yonsei.ac.kr (J.H.); chihyun.park@yonsei.ac.kr (C.P.); 2 Department of Computer Science, University of Illinois at Urbana-Champaign, Urbana, OH 61801, USA; pcy1302@illinois.edu

**Keywords:** miRNA, disease, miRNA-disease association, miRNA similarity network, matrix factorization

## Abstract

The identification of potential microRNA (miRNA)-disease associations enables the elucidation of the pathogenesis of complex human diseases owing to the crucial role of miRNAs in various biologic processes and it yields insights into novel prognostic markers. In the consideration of the time and costs involved in wet experiments, computational models for finding novel miRNA-disease associations would be a great alternative. However, computational models, to date, are biased towards known miRNA-disease associations; this is not suitable for rare miRNAs (i.e., miRNAs with a few known disease associations) and uncommon diseases (i.e., diseases with a few known miRNA associations). This leads to poor prediction accuracies. The most straightforward way of improving the performance is by increasing the number of known miRNA-disease associations. However, due to lack of information, increasing attention has been paid to developing computational models that can handle insufficient data via a technical approach. In this paper, we present a general framework—improved prediction of miRNA-disease associations (IMDN)—based on matrix completion with network regularization to discover potential disease-related miRNAs. The success of adopting matrix factorization is demonstrated by its excellent performance in recommender systems. This approach considers a miRNA network as additional implicit feedback and makes predictions for disease associations relevant to a given miRNA based on its direct neighbors. Our experimental results demonstrate that IMDN achieved excellent performance with reliable area under the receiver operating characteristic (ROC) area under the curve (AUC) values of 0.9162 and 0.8965 in the frameworks of global and local leave-one-out cross-validations (LOOCV), respectively. Further, case studies demonstrated that our method can not only validate true miRNA-disease associations but also suggest novel disease-related miRNA candidates.

## 1. Introduction

MicroRNAs (miRNAs) are small single-stranded non-coding RNAs that bind to the 3′ untranslated regions (UTRs) of target messenger RNAs (mRNAs) [1,2]. miRNAs tend to restrain gene expression by control of their own regulatory sequences and promoters; they bind to specific target mRNAs through base-paring, which inhibits the translation and stability. Since the first discovery of two miRNAs (Caenorhabditis elegans lin-4 and let-7) in 1993 and 2000, increasing attention has been paid to this research field. Numerous studies continue to demonstrate the crucial roles of miRNAs in diverse biologic processes such as apoptosis [3], cell development [4], proliferation [5], viral infection [6] and metabolism [7]. As indicated by previous studies, miRNAs are becoming diagnostic/therapeutic tools for diseases as well as potential prognostic biomarkers. For example, lower expression of miR-195 appeared in Alzheimer’s disease (AD) patients [8] and miR-101 was shown to be a significant factor in breast cancer by targeting Stathmin1 [9]. Furthermore, miR-15 and miR16 were deleted in more than half of the cases of B-cell chronic lymphocytic leukemia (B-CLL) [10]. Experiments further validated that miR-185 plays a crucial role in breast cancer by targeting Vegfa [11] and miR-122 inhibits cell proliferation and tumorigenesis of breast cancer by targeting IGF1R [12]. Therefore, predicting miRNA-disease associations can expand the understanding of molecular mechanisms of multiple human diseases and novel prognostic biomarkers.

Considering the time and costs involved in wet experiments, predicting disease-related miRNAs through in silico experiments can be a good alternative while enhancing the prediction accuracy. To this end, increasing attention has been paid to the design of competitive and effective computational models to explore novel miRNA-disease associations. According to recent studies, existing computational models can be mainly categorized into two categories: similarity-based and machine-learning-based models. Similarity-based models predict novel disease-related miRNAs based on the assumption that functionally similar miRNAs have a high possibility to be involved in phenotypically similar diseases and vice versa. Machine-learning-based models predict miRNA-disease associations by adjusting the optimal parameter combination of the model.

Jiang et al. developed a miRNA-disease association prediction model by integrating the miRNA functional similarity network, disease similarity network and phenome-microRNAome network [13]. Mork et al. developed the computational model of miRPD that utilizes experimentally verified miRNA-protein interactions and text-mined results of protein-miRNA interactions to indirectly determine the miRNA-disease association [14]. In miRPD, proteins play a significant role as mediators to link the miRNA-disease associations. Hence, miRNA-disease associations with more commonly shared proteins are more likely to have high scores in miRPD. However, this method is biased towards protein links, which is not applicable to the miRNAs with no protein interactions, thereby limiting further improvement. Chen et al. proposed the similarity-based model of random walk with restart for miRNA-disease association (RWRMDA) [15]. The authors first assigned the initial probability on each node of the miRNA functional similarity network (MFSN); the random walk algorithm was implemented before the probability of each node became stable. However, this model is not suitable for the miRNAs with no disease associations; it leads to poor prediction accuracy. They also proposed a prediction framework called within and between score for miRNA-disease association prediction (WBSMDA) [16]. WBSMDA was developed to uncover the potential link between miRNAs and complex human diseases by applying miRNA functional similarity, disease semantic similarity and Gaussian interaction profile kernel similarity of miRNAs and diseases. This can be applied to new miRNAs and diseases without any prior information. Chen et al. further investigated the prediction of known disease-related miRNAs by presenting a computational model—heterogeneous graph inference for miRNA-disease association prediction (HGIMDA) [17]. HGIMDA integrated the miRNA functional similarity, disease semantic similarity and Gaussian interaction profile kernel similarity to successfully reveal disease-related miRNAs by exploring all three-length paths in the heterogeneous network. Xuan et al. proposed the computational model of human disease-related miRNA prediction (HDMP) that predicts novel miRNA-disease associations by considering the weighted k most similar neighbors [18]. HDMP assigns more weight to miRNAs within the same miRNA family or cluster. However, the chosen number of k-nearest neighbors highly affects the prediction performance and leaves room for improvement in accuracy by making full use of global network information.

With the rapidly growing amount of information available through various in vivo experiments, it has become inevitable to inject the auxiliary omics datasets into the prediction model for discovering novel miRNA-disease associations. Owing to the aid of the diverse biologic data, various computational prediction models enhanced the prediction accuracy by prioritizing the disease-related miRNAs in terms of prediction scores, which were assigned by each model. Ha et al. proposed the similarity-based network model to predict the potential miRNA-disease associations [19]. They measured the similarity among miRNAs based on the assumption that two miRNAs are functionally related if the number of shared environmental factors is statistically significant. Environmental factors include drugs, alcohol, stress and diet. However, this model does not consider the chemical structure of the EF, which leaves a room for improvement in prediction accuracy by measuring the precise similarity among the miRNAs. Shi et al. made use of the protein-protein interaction (PPI) network by implementing the random walk algorithm to exploit miRNA-disease associations [20]. In summary, most similarity-based models have encountered difficulties in their performance owing to the lack of sufficient validated interactions. Hence, these approaches are highly biased towards miRNA-disease associations, which is not applicable for the miRNAs with no disease associations.

Machine learning-based approaches have delivered superior performance in various scientific research areas including bioinformatics and computational biology. For example, Chen et al. developed the computational framework named regularized least square for miRNA-disease association (RLSMDA) [21]. This study is based on semi-supervised learning that can predict miRNA-disease associations without using negative samples. However, the main drawback of this model is finding optimal parameters of RLSMDA and combining the classifiers from two different spaces. Chen et al. also presented the model of the restricted Boltzmann machine for multiple types of miRNA-disease prediction (RBMMMDA) by utilizing the restricted Boltzmann machine (RBM) [22]. The main advantage of this model is not just the resulting improvement in prediction accuracy, but mainly the ability of estimating the corresponding types of miRNA-disease associations. Ha et al. proposed the matrix factorization-based model called PMAMCA to identify potential miRNA-disease associations [23]. This model, with the utilization of miRNA expression data and known miRNA-disease associations, outperformed the previous models in terms of area under the receiver operating characteristic (ROC) curve (AUC) scores. However, this model leaves room for further improvement by using diverse biologic information as implicit data. To date, Li et al. proposed a matrix completion algorithm-based model called MCMDA [24]. In this study, they constructed binary adjacency matrix with known miRNA-disease associations, and a singular value threshold (SVT) algorithm was constructed to find novel disease-related miRNAs. However, finding optimal parameters of this model remains a critical issue. Xio et al. proposed the framework graph regularized non-negative matrix factorization (GRNMF) that exploits the weighted gene network to calculate the interaction profiles of new miRNAs and diseases [25]. Chen et al. developed a model of ranking-based k-nearest neighbors for miRNA-disease association prediction (RKNNMDA) by exploring the k-nearest-neighbors of miRNAs and diseases. SVM was adopted for calculating the k-nearest-neighbors, and prioritized miRNA-disease associations based on weighted voting [26]. Chen et al. proposed the approach of inferring miRNA-disease associations by making complete use of inductive matrix complementation with matrix decomposition and heterogeneous graphs (IMCMDA) [27]. This model not only explores disease-related miRNAs but also measures the comprehensive similarities of miRNAs and diseases. Chen et al. presented the matrix decomposition and heterogeneous graph inference (MDHGI). This model prioritizes disease-related miRNAs by combining the matrix decomposition algorithm with miRNA functional similarity, disease semantic similarity and Gaussian interaction profile kernel similarity [28]. In summary, most machine learning-based approaches have difficulties in adjusting the optimal parameters and using negative samples. Furthermore, various optimal parameter combinations may exist in different scenarios, thereby resulting in more complicated sensitivity analysis.

Identifying novel miRNA-disease associations is beneficial for the understanding of disease pathogenesis at the molecular level and the development of the disease diagnostic biomarkers. However, most previous miRNA-prediction algorithms are still impeded by the data sparsity problem; hence, it is challenging to predict the miRNAs with a few known disease associations. These miRNAs are called rare miRNAs. In the recommender system, similar problems were efficiently addressed by adopting matrix factorization to predict the most plausible rating scores of each user. Inspired by the recent advancement of recommender systems, this study addresses common research problems by formalizing a matrix factorization-based model for collaborative filtering. In the light of this issue, we transform the task of predicting miRNA-disease associations in a recommender task.

In this study, we present a computational miRNA-disease association prediction model for improved prediction based on matrix completion with network regularization (IMDN). We consider the miRNA network to efficiently handle rare miRNAs. The core idea of IMDN is based on the consideration of relationship among the miRNAs within the network to better capture the embeddings through the direct neighbors. To inject the influence of miRNA network, we coin the network regularization term to consider network constraints on the prediction model. Because of the limited number of predetermined weight values on the miRNA similarity network, our proposed model was extended to calculate the precise miRNA similarity through the Gaussian interaction profile kernel. Our primary contribution to IMDN relies on its expandability of matrix factorization-based model, which applies miRNA similarity network as the regularization term and miRNA expression value as the weight of the objective function. By mapping the miRNA expression value as a weight of the objective function, we could train the model even though we do not know the miRNA-disease associations. Further, calculation of new similarities among the miRNAs could be one of the main contributions to the delivery of outstanding performance. We expect that IMDN can serve as an effective tool for discovering potential miRNAs-disease associations by considering the miRNA network. Various experimental results demonstrated that IDMN outperforms the state-of-the-art miRNA-disease association prediction model in terms of the AUC scores and the survival analysis.

## 2. Materials and Methods

### 2.1. Methods Overview

**Figure 1:**
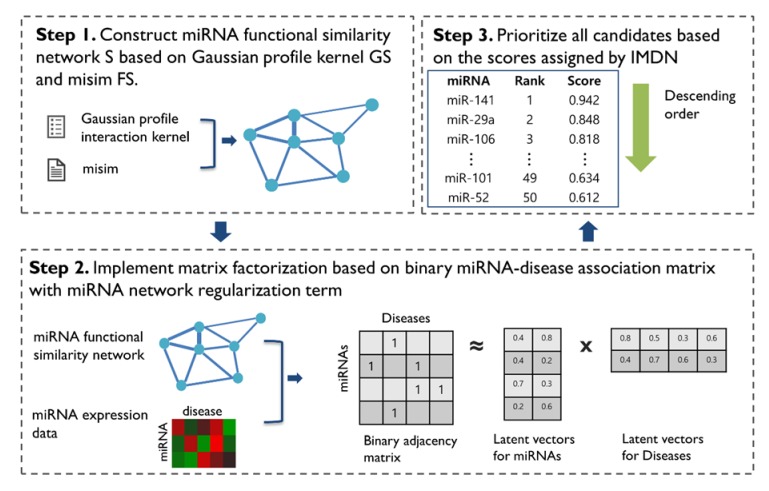
Workflow of IMDN. First, the functional similarity network in which node is miRNA was constructed from already known database misim and the proposed inference approach. Second, matrix factorization was performed with both the inferred miRNA network and miRNA expression data. Finally, prioritization was implemented based on the highly scored miRNAs.

We present the novel computational framework of IMDN to predict miRNA-disease associations. IDMN comprises three main steps. First, to construct the miRNA functional similarity network, we utilize the pre-calculated weight of misim and calculate the new miRNA similarity through the Gaussian interaction profile kernel. Second, given a miRNA similarity network and miRNA expression data, we apply matrix factorization-based model to efficiently train the miRNA latent feature vector and disease latent feature vector based on the known miRNA-disease associations. Lastly, we prioritize miRNA candidates based on scores that were assigned by the IDMN. The workflow of IDMN is illustrated in detail in Figure 1.

### 2.2. Human miRNA-disease Associations

We collected human miRNA-disease associations data from HMDD v2 [29], dbDEMC [30] and miR2Disease [31]. Despite the comparable effectiveness of MF in a wide variety of domains, the challenge in prediction performance remains owing to the insufficient experimentally validated interactions in binary adjacency matrix R. Therefore, the operation of combining the miRNA-disease associations from three public databases was conducted to produce rich input data. As duplicate entries exist in the three public databases, we implemented data preprocessing to eliminate the duplicates. HMDD is an online public database that provides 10,368 experimentally confirmed human miRNA-disease associations regarding 572 miRNAs and 378 diseases. dbDEMC is an integrated human miRNA database of differentially expressed miRNAs in human cancers (dbDEMC) that contains information on 2224 miRNAs and 36 diseases. miR2disease is a manually curated database that contains in the form of 1939 entries on 299 miRNAs and 94 diseases. After unification, we conducted an operation unifying the names of different miRNAs under one miRNA gene based on standard mesh disease terms. Variables $N_{m}\;$ and $N_{d}$ stand for the number of miRNAs and diseases, respectively, and the $N_{m}$ × $N_{d}$ binary adjacency matrix R was constructed on the basis of the integrated human miRNA-disease associations. The binary adjacency matrix R is expressed as follows:

(1) $$R(m(u),d(i))=\{\begin{array}{l} 1,\;if\;miRNA\;m\left(u\right)\;and\;disease\left(i\right)has\;verified\;association\;\\ 0,\;otherwise \end{array}$$

### 2.3. miRNA Expression Data

To model the prediction model more precisely and effectively, we utilized the miRNA expression dataset to compensate insufficient miRNA-disease associations. As a large number of biologic datasets are being generated with the help of the high-throughput technique, these datasets create opportunities to decipher the understanding of diverse meaningful biologic functions such as disease pathogenesis and disease etiology as well as discover novel disease biomarkers. Therefore, miRNA expression data were obtained from the cancer genome atlas (TCGA), which provides multimodal genomics and proteomics data for thousands of tumor samples for more than 20 types of cancer [32]. To construct the $N_{m}$ × $N_{d}$ miRNA expression weight matrix W, min-max normalization was conducted first. We only take the weight value W (u,i) into account when there is no association between miRNA m(u) and disease d(i) in the original matrix R, otherwise, we regard it as one.

(2) $$w_{ui}=\{\begin{array}{ll} 1 & if\;R_{ui}=1\\ miRNA\;expression\;value & if\;R_{ui}=0 \end{array}$$

### 2.4. miRNA Similarity Network

#### 2.4.1. miRNA Functional Similarity

miRNA functional similarity scores were calculated based on the hypothesis that functionally similar miRNAs are more inclined to associate with phenotypically similar diseases. miRNA Functional similarity data misim 2.0 was downloaded from http://www.lirmed.com/misim/ to construct the $N_{m}$ × $N_{m}$ miRNA functional similarity matrix FS [33]. The similarity score between miRNA m(u) and m(i) can be expressed as FS(u,i).

#### 2.4.2. Gaussian Interaction Profile Kernel miRNA Similarity

Multiple studies continue to prove the effectiveness of the Gaussian interaction profile kernel on calculating similarities among both diseases and miRNAs [34,35]. To calculate the comprehensive and precise similarity score among the miRNAs, we adopted the Gaussian kernel function, which is also called radical basis function (RBF). We regraded two miRNAs to be functionally related if they have similar patterns of interactions with the diseases on the basis of the known human miRNA-disease associations. For a given miRNA u, the feature vectors of IP(m(u)) were extracted from the i-th row of the miRNA latent feature vector to express the interaction profile of m(u). The Gaussian kernel similarity between miRNA m(i) and m(j) could be computed by:

(3) $$\mathrm{GS}\left(m\left(u\right),\;m\left(i\right)\right)=\exp(-r_{m}{\left|\left|\;IP\left(m\left(u\right)\right)-IP\left(m\left(i\right)\right)\right|\right|}^{2})$$

GS is denoted as Gaussian interaction profile kernel, where $r_{m}^{'}$ is the hyperparameter that controls the bandwidth of the kernel, which can be calculated as follows:

(4) $$r_{m}=\frac{r_{m}^{'}}{\frac{1}{n_{m}}\sum_{i=1}^{n_{m}}||IP(m\left(u\right)|{|}^{2}}$$

#### 2.4.3. Integrated miRNA Similarity

We obtained the integrated miRNA similarity score that was used for constructing miRNA similarity network based on the miRNA functional similarity FS and miRNA Gaussian interaction kernel similarity GS. The integrated weight value that was used for the edge of miRNA similarity network S can be expressed as follows:

(5) $$S\left(m\left(u\right),m\left(i\right)\right)=\{\begin{array}{l} FS\left(m\left(u\right),m\left(i\right)\right)\;if\;m\left(u\right)and\;m\left(i\right)has\;functional\;simialrity\\ GS\left(m\left(u\right),m\left(i\right)\right)\;otherwise \end{array}$$

### 2.5. IMDN

Among various collaborative filtering methods, matrix factorization has yielded immense success on recommendation systems [36]. However, the large-scale and sparse data of the original matrix usually degrades the performance of the matrix factorization model. Hence, most of the matrix factorization-based models are suffering from a cold start problem when there are miRNAs with few disease associations in the binary adjacency matrix. To handle this issue, various advanced matrix factorization methods have been proposed by utilizing various biologic datasets. In this work, we used the miRNA network as auxiliary information to enhance the prediction accuracy.

The miRNA network can be defined as a graph where there is a node corresponding to each miRNA, and an edge corresponding to each similarity weight. The physical meaning of the weight edge in network $S_{u,i}$ can be interpreted as how much miRNA $M_{u}$ is similar to the miRNA $M_{i}$.

Applying the network influence, the trait of each miRNA can be affected by its direct neighbors $E_{u}$. Based on the intuition that nodes have similar structural roles in network should be located close together, the miRNA latent feature vector $M_{u}$ is highly affected by the latent feature vectors of its direct neighbors $v\in E_{u}$. ${\hat{M}}_{u}$ is an estimated latent feature calculated from feature vectors of its direct neighbors. All the notations, which were used in following equations, are described in Table 1. Formulation is described as follows:

(6) $${\hat{M}}_{u}=\frac{\sum_{v\in E_{u}}S_{u,v}M_{v}}{\sum_{v\in E_{u}}S_{u,v}}=\frac{\sum_{v\in E_{u}}S_{u,v}M_{v}}{\left|E_{u}\right|}$$

By fully taking advantage of the characteristic of miRNAs in the miRNA similarity network, the new estimated latent feature vector of miRNA can be calculated by the weighted average of its direct miRNA latent feature vectors as follows:

(7) $$\left(\begin{array}{l} {\hat{M}}_{u,1}\\ {\hat{M}}_{u,2}\\ \ldots \\ {\hat{M}}_{u,k} \end{array}\right)=\left(\begin{array}{llll} M_{1,1} & M_{2,1} & \ldots & M_{N,1}\\ M_{1,2} & M_{2,2} & \ldots & M_{N,2}\\ \ldots & ... & \ldots & \ldots \\ M_{1,k} & M_{2,k} & \ldots & M_{N,k} \end{array}\right)\left(\begin{array}{l} S_{u,1}\\ S_{u,2}\\ \ldots \\ S_{u,N} \end{array}\right)$$

Considering the miRNA similarity network as implicit feedback does not change the conditional distribution of known miRNA-disease associations. It only takes miRNA latent vectors into account. Therefore, the expression of conditional probability can be expressed as follows.

(8) $$p\left(R|M,D,\;\sigma_{R}^{2}\right)=\prod_{u=1}^{N_{m}}\prod_{i=1}^{N_{d}}{\left[N(R_{u,i}|g\left(M_{u}^{T}D_{i}\right),\sigma_{R}^{2})\right]}^{I_{u,i}^{R}}$$

The zero-mean Gaussian prior is assigned to miRNA latent vectors to avoid over-fitting. Motivated by the fact that characteristic of miRNA is highly affected by its direct neighbor, conditional distribution of miRNA latent vector is given the latent vectors of its direct neighbors as follows:

(9) $$p(M,D|R,S,\;\sigma_{R}^{2},\;\sigma_{S}^{2},\sigma_{M}^{2},\sigma_{D}^{2})\;\propto p(R|M,D,\;\sigma_{R}^{2})\;p(M|S,\;\sigma_{M}^{2},\;\sigma_{S}^{2})\;p\left(D|\sigma_{D}^{2}\right)=\prod_{u=1}^{N_{m}}\prod_{i=1}^{N_{d}}{\left[N(R_{u,i}|g\left(M_{u}^{T}D_{i}\right),\sigma_{R}^{2})\right]}^{I_{u,i}^{R}}\times \prod_{u=1}^{N_{m}}N(M_{u}|\underset{v\in E_{u}}{\sum }S_{u,v}M_{v},\sigma_{S}^{2}I)\times \prod_{u=1}^{N_{m}}N(M_{u}|0,\sigma_{M}^{2}I)\times \prod_{i=1}^{N_{d}}N(D_{i}|0,\sigma_{D}^{2}I)$$

Our goal is to capture the most plausible latent vectors of miRNAs $M_{u}\;$ and diseases $D_{i}$, so that the inner product of each latent vector would be close to the entry of binary association matrix $R_{u,i}$. Aiming at modeling the cost function more accurately, we added additional miRNA terms to better capture the characteristic of miRNA latent vector $M_{u}$ which naturally reflects the neighbors’ characteristic of $M_{v}$ in the miRNA similarity network S. We also coin the miRNA expression weight matrix as W to efficiently train the latent vector of miRNA and disease.

(10) $$lnp(M,D|R,S,\;\sigma_{R}^{2},\;\sigma_{S}^{2},\sigma_{M}^{2},\sigma_{D}^{2})=-\frac{1}{2\sigma_{R}^{2}}\sum_{u=1}^{N_{m}}\sum_{i=1}^{N_{d}}W_{u,i}{\left(R_{u,i}-g\left(M_{u}^{T}D_{i}\right)\right)}^{2}-\frac{1}{2\sigma_{M}^{2}}\sum_{u=1}^{N_{m}}M_{u}^{T}M_{u}-\frac{1}{2\sigma_{D}^{2}}\sum_{i=1}^{N_{d}}D_{i}^{T}D_{i}-\frac{1}{2\sigma_{S}^{2}}\sum_{u=1}^{N_{m}}\left({\left(M_{u}-\sum_{v\in E_{u}}S_{u,v}M_{v}\right)}^{T}\left(M_{u}-\sum_{v\in E_{u}}S_{u,v}M_{v}\right)\right)-\frac{1}{2}\left(\sum_{u=1}^{N_{m}}\sum_{i=1}^{N_{d}}W_{u,i}^{R}\right)ln\sigma_{R}^{2}-\frac{1}{2}\left(\left(N_{m}\times N_{l}\right)ln\sigma_{M}^{2}+\left(N_{d}\times N_{l}\right)ln\sigma_{D}^{2}+\left(N_{m}\times N_{l}\right)ln\sigma_{S}^{2}\right))+C$$

Maximizing the log-posterior over latent vectors of miRNAs and diseases can be thought of equivalent to minimizing the cost function below. The goal is to minimize the loss between the entry of $R_{u,i}$ and dot product of corresponding miRNA latent vector $M_{u}$ and disease latent vector $D_{i}$.

(11) $$L\left(R,S,M,D\right)=\frac{1}{2}\sum_{u=1}^{N_{m}}\sum_{i=1}^{N_{d}}W_{u,i}{\left(R_{u,i}-g\left(M_{u}^{T}D_{i}\right)\right)}^{2}+\frac{\lambda_{M}}{2}\sum_{u=1}^{N_{m}}M_{u}^{T}M_{u}+\frac{\lambda_{D}}{2}\sum_{i=1}^{N_{d}}D_{i}^{T}D_{i}+\frac{\lambda_{S}}{2}\sum_{i=1}^{N_{m}}({\left(M_{u}-\underset{v\in E_{u}}{\sum }S_{u,v}M_{u}\right)}^{T}\left(M_{u}-\underset{v\in E_{u}}{\sum }S_{u,v}M_{u}\right))$$

**Figure 2:**
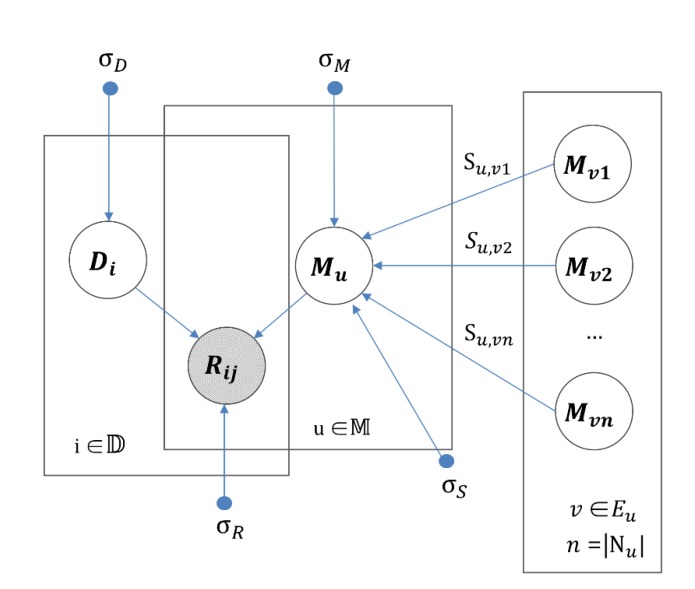
Graphical modeling of IMDN.

The derivative of $M_{u}$ and $D_{i}$ for all miRNAs u and all diseases *i* can be expressed as follows by performing a gradient decent. Our approach is efficient even when performing a simple gradient descent method. $\lambda_{M}$**,** $\lambda_{D}$**,** $\lambda_{S}$ are the hyper-parameters that were applied to control regulators to avoid overfitting. Graphical modeling of IMDN is illustrated in Figure 2.

(12) $$\frac{\partial ℒ}{\partial M_{u}}=\sum_{u=1}^{N_{m}}W_{u,i}D_{i}g^{\prime }\left(M_{u}^{T}D_{i}\right)(\;g\left(M_{u}^{T}D_{i}\right)-R_{u,i})+\lambda_{M}M_{u}+\lambda_{S}(M_{u}-\sum_{v\in E_{u}}S_{u,v}M_{u})-\lambda_{S}\sum_{\left\{v|u\in E_{v}\right\}}S_{v,u}(M_{v}-\sum_{u\in E_{v}}S_{v,w}M_{w})$$

(13) $$\frac{\partial ℒ}{\partial D_{i}}=\sum_{u=1}^{N_{d}}W_{u,i}M_{u}g^{\prime }(M_{u}^{T}D_{i})(g\left(M_{u}^{T}D_{i}\right)-R_{u,i})+\lambda_{D}D_{i}$$

## 3. Results

### 3.1. Performance Evaluation

To demonstrate the superiority of IMDN, we compared our method with other state-of-the-art methods such as PMAMCA [23], MDHGI [28], RKNNMDA [26], RWRMDA [15], MCMDA [24] and RLSMDA [21]. All models were assessed by implementing leave-one-out cross-validation (LOOCV) based on integrated miRNA-disease associations (dbDEMC, miR2diseaes and HMDD v2). Typically, LOOCV can be divided into global and local LOOCV, wherein each known miRNA-disease association was left out in turn as a test sample, whereas all the other remaining miRNA-disease pairs were considered as training samples. Global LOOCV evaluates the performance of the model by considering all diseases simultaneously, whereas local LOOCV only considers miRNAs for a specific disease. That is to say, in global LOOCV, each association was considered as test sample while in turn the remains were regarded as training samples. In local LOOCV, assessment of local prediction was performed by considering the ability to recover the miRNA-disease associations for a specific disease.

For both global and local LOOCV, all test samples are prioritized based on the prediction scores assigned by IMDN. This partition-prediction-ranking step was conducted 100 times to derive the mean AUC score of IMDN for reasonable estimation of the prediction accuracy. The AUC scores were calculated to demonstrate the performance of each method. We drew the ROC curve in terms of the true positive rate (TPR, sensitivity) and false positive rate (FPR, 1-specificity), where sensitivity and specificity could be defined as follows:

(14) $$Sensitivity=\frac{TP}{TP+FN}$$

(15) $$Specificity=\frac{TN}{TN+FP}$$

**Figure 3:**
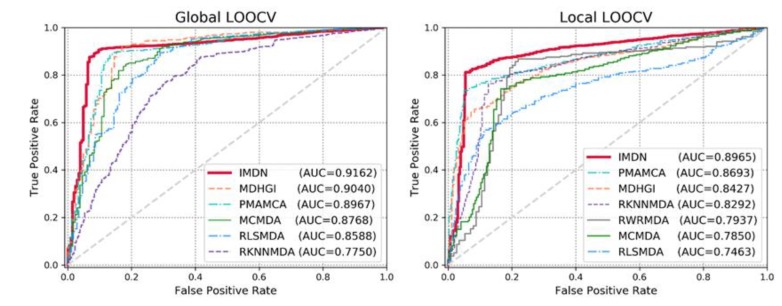
Performance comparisons based on global local leave-one-out cross-validations (LOOCV) and local LOOCV in terms of area under the curve (AUC) scores. As is shown, IMDN achieved AUCs of 0.9162 and 0.8965, respectively, outperforming the previous models.

**Figure 4:**
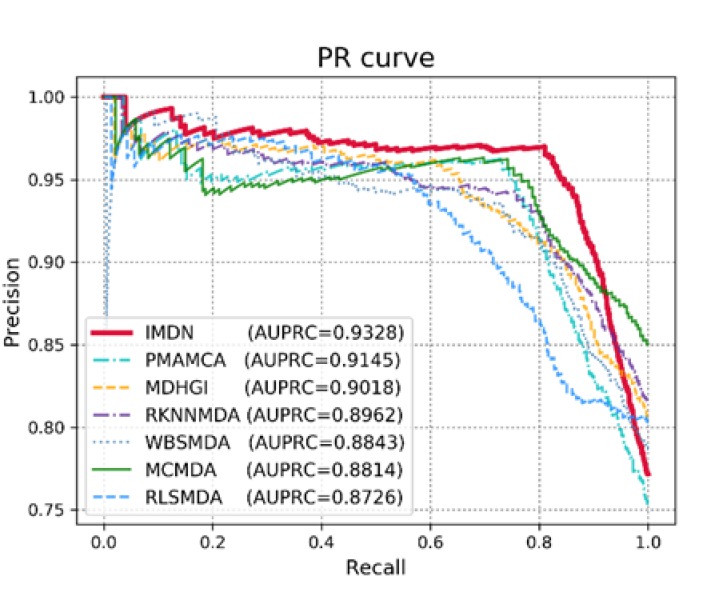
Performance comparisons based on Precision/Recall curve in terms of AUPRC scores.

Sensitivity refers to the extracted candidates ranked above the threshold and specificity refers to the candidates that are ranked below the threshold. TP and TN denote the numbers of correctly identified positive and negative samples, whereas FP and FN denote the numbers of misidentified positive and negative samples. Typically, an AUC value of 1 represents perfect prediction, whereas an AUC value of 0.5 represents random selections. Therefore, models with AUC scores that are close to 1 are considered competitive prediction models. We demonstrate the efficacy of IDMN over state-of-the-art methods by comparing the AUC scores. The performance comparison in terms of the ROC curve is illustrated in Figure 3. As shown in Figure 3, IMDN obtained an AUC value of 0.9162 in global LOOCV, which is superior to MDHGI (0.9040), PMAMCA (0.8967), MCMDA (0.8768), RLSMDA (0.8588) and RKNNMDA (0.775). As for local LOOCV, IMDN obtained an AUC value of 0.8965, which is superior to PMAMCA (0.8693), MDHGI (0.8427), RKMFMDA (0.8292), RWRMDA (0.7937), MCMDA (0.7850) and RLSMDA (0.7463). RWRMDA was not able to perform comparison evaluation based on global LOOCV because it considers diseases one at a time. To demonstrate the performance of IDMN more precisely, we additionally drew precision/recall curve and calculated auprc scores. As illustrated in Figure 4, IMDN achieved the best performance compared to previous prediction models. The comparison shows that IMDN achieves a comparable performance under the reliable evaluation metric, which supports that our approach is capable of predicting a large number of disease-related miRNAs.

### 3.2. Effect of miRNA Functional Similarity Network

**Figure 5:**
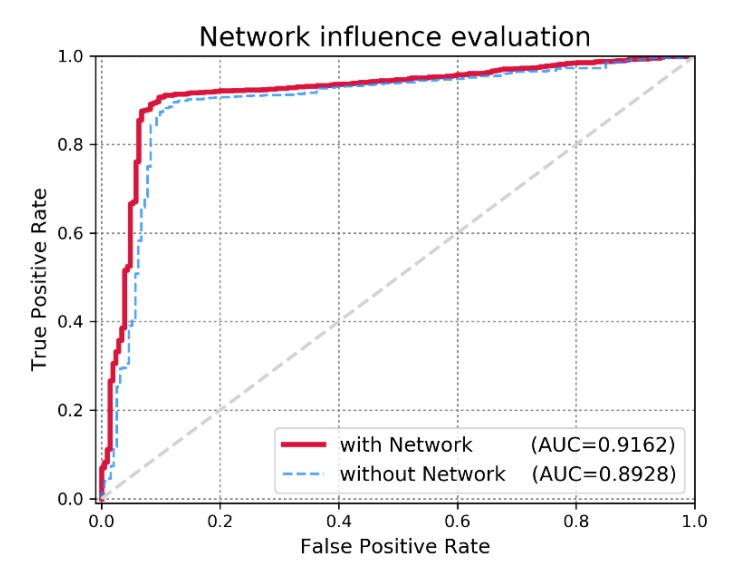
Performance evaluation on IMDN with the miRNA functional similarity network and without the miRNA functional similarity network.

With the vast sizes of biologic datasets that are generated nowadays, an important issue for evaluating IDMN is whether the model efficiently reflects additional biologic data. We validated the possible expandability of IDMN for the new input data (i.e., implicit feedback) such as miRNA functional similarity network data. In this study, we used the network regularization term to inject the information of miRNA functional similarity data into the matrix factorization-based model. To demonstrate the efficacy of miRNA functional similarity information, we checked the prediction accuracy in two cases: 1) without the network regularization term, we only mine the miRNA-disease association binary matrix and employ known miRNA-disease associations for making predictions; 2) with the network regularization term, we fuse the information from the miRNA similarity graph to capture the trait of each miRNA purely from its direct neighbors. Consequently, we could confirm the significant increase in the performance of IDMN with the miRNA functional similarity network, as illustrated in Figure 5. The motivation behind applying the miRNA similarity network was to reflect the hidden characteristics through its direct neighbors. We can conclude that IDMN supports the well-known biologic assumption that functionally similar miRNAs are inclined to associate with phenotypically similar diseases.

### 3.3. Case Studies

We also studied three main common diseases in the human population to qualitatively ascertain the performance of IMDN for novel disease-related miRNA prediction. We observed the number of correctly identified disease-related miRNAs for the three diseases within the top 50 candidates.

Colon neoplasm (CN) is the most common malignant cancer that typically arises from lesions in the human colon or rectum, which poses a major threat to human life. According to the latest statistic in 2019 [37], 145,600 newly diagnosed CN cases and 51,020 deaths from CN were reported in the United States. To date, many researchers have proposed that the utilization of miRNAs as new biomarkers can be a good alternative for detecting CN. Therefore, IMDN was implemented to predict the potential CN-related miRNAs by prioritizing the candidates with the scores assigned by IMDN. As shown in Table 2, IMDN confirmed 46 out of the top 50 CN-related miRNAs. Among the four remaining candidates, three were validated by experimental studies. For example, miR-150 was found to function as a tumor suppressor in CN by targeting c-Myb [38]; overexpression of miR-122 could lead to the development of CN liver metastasis [39]; expression of miR-199a-3p (pre-miRNA of miR-199a) could be involved in the development, tumorigenesis and progression of CN [40]. Consequently, 49 out of the top 50 potential CN-related miRNAs were validated by experimental results.

Kidney neoplasm (KN) is a nonhomogeneous cancer that accounts for 5% of the new male cancer cases. Approximately 73,820 new KN cases were reported in the United States in 2019 [37]. Recent studies showed that miRNAs can play a role in discovering the hidden mechanism of KN. Therefore, we applied IDMN to extract potential miRNAs that are relevant to KN. As shown in Table 3, 46 out of the top 50 candidates were confirmed to be KN-related miRNAs, whereas the remaining four candidates were validated by recent studies. Overexpression of miR-142–3p could induce the apoptosis in RCC 786-O and ACHN cells. RCC is the most common type of adult kidney cancer [41]. Expression of miR-30a-5p was found to be substantially downregulated in the RCC tissues compared to normal tissues [42]. To conclude, 48 out of the top 50 proved to be KN-related miRNAs by public databases and other publications.

Lymphoma is a malignant tumor that has its origin in a type of white blood cells called lymphocytes. Lymphoma can be divided into two main types: Hodgkin lymphoma (HL) and non-Hodgkin lymphoma (NHL) [43]. According to statistics, 90% of people with lymphoma have non-Hodgkin’s lymphoma [44]. Recently, to elucidate the pathogenesis of lymphoma, researchers proposed the miRNAs as a novel biomarker. Experimental studies demonstrated that deletion or down-regulation of miR-15a leads to overexpression of B cell lymphoma 2 (BCL2), which is a common phenomenon of Lymphoma [45]. Moreover, studies have shown that overexpression of miR-18b may help in identifying patients with poor prognosis in cell lymphoma treated cohorts. [46]. Therefore, it is imperative to take lymphoma as a case study to verify the prediction performance. After implementing IMDN for lymphoma as a case study, we confirmed that 45 out of 50 candidates proved to be lymphoma-related miRNAs. Table 4 shows this result.

### 3.4. Survival Analysis

**Figure 6:**
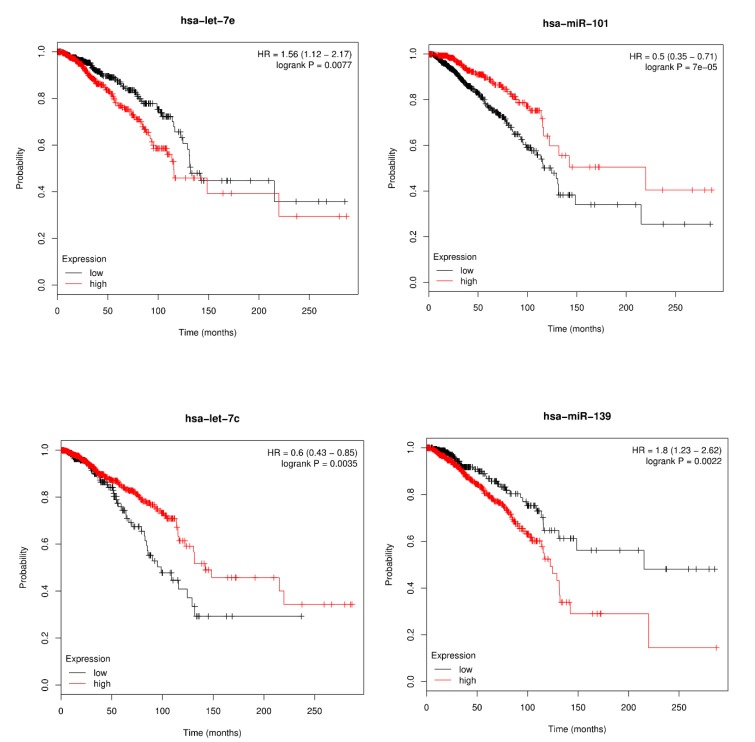
Kaplan-Meier plots of hsa-miR-148a, hsa-miR-133b, hsa-miR-130a and hsa-let-7e for survival of breast cancer patients.

Consideration of the relationship between miRNAs and prognosis of breast cancer can give new insights into disease etiology [47,48]. We analyzed whether miRNA, which was identified to be related with certain disease, could be used as a prognostic biomarker according to the change in expression level. We also performed survival analysis by plotting Kaplan-Meier curve and testing statistical significance based on log-rank test. miRpower-Kaplan-Meier plotter web tool provides the function of Kaplan-Meir survival analysis [49]. We only considered the miRNAs with a *p*-value less than 0.005 as significant when factoring the overall survival rate of breast cancer patients. By performing the Kaplan-Meier survival analysis of the highly ranked miRNA candidates (has-let-7e, has-miR-101, has-let-7c and has-miR-139), we could prove that these miRNAs highly associate with the survival rates of breast cancer patients. The overall analysis is illustrated in Figure 6.

## 4. Discussion

Identification of potential miRNA-disease associations could expand the understanding of disease etiology and pathogenesis. To this end, this study presents the novel framework of improved prediction of miRNA-disease associations based on matrix completion with network regularization (IMDN) for prioritization of disease-related miRNAs. The goal of IMDN is to learn miRNA and disease latent vectors through matrix factorization while preserving the properties of miRNA-disease associations. With the vast amount of omics datasets that are publicly available, an important criterion for evaluating the IMDN is whether the model effectively reflects additional biologic data while enhancing the prediction accuracy. Traditional MF-based prediction models are highly dependent on the known miRNA-disease associations while they ignore the relationship among the miRNAs in the network. To address this issue, we modified a cost function that we could use to adaptively learn miRNAs and disease latent vectors, given the miRNA similarity network constructed using misim and Gaussian interaction profile kernels. Our prediction model was characterized by fully exploring the constructed miRNA similarity network to inject the correlations among the miRNAs. After implementing matrix factorization model with various biologic data, it was natural that miRNAs with a high chance of involvement in disease incidence would be highly prioritized with a high score. The AUC value was adopted to measure the prediction accuracy. As a result, the IMDN delivered superior performance with reliable AUC values of 0.9162 and 0.8965 in the frameworks of global and local LOOCV, respectively. Furthermore, case studies were conducted on three significant human diseases to verify the stable and reliable performance of IMDN. In summary, the experiments under various evaluation metrics qualitatively validated the excellent performance of IMDN compared to previous methods.

## 5. Conclusions

The excellent prediction performance of IDMN may be attributed to several important factors. First, we applied a matrix factorization model that yielded immense success in the recommender system. Among various collaborative filtering techniques, matrix factorization has been a promising technique in a wide variety of domains. In bioinformatics, matrix factorization helps in identifying hidden links among genes—and in recommender systems—it infers the most plausible rating scores that users may give to certain items. Thus, we transform the prediction of miRNA-disease associations into a recommender task. Second, IMDN is expandable in terms of additional biologic data, such as miRNA expression data and it improves the prediction accuracy. Lastly, our model exploited not only the known miRNA-disease associations but also integrated the miRNA similarity to better capture the characteristic of miRNA through its direct neighbors in the miRNA similarity network. It is noteworthy that the consideration of the miRNA similarity network lead to train the miRNA latent vector well. Most importantly, we anticipated that IMDN can serve as an effective tool for discovering potential links between miRNAs and diseases.

For future work, larger biologic datasets can be used to better capture the latent vectors of miRNAs and diseases to infer potential disease-related miRNAs. Furthermore, evaluation of miRNA candidates with not only the in silico experiments but also in vivo experiments shall clearly demonstrate the performance of the model and improve the credibility of the study. We also expect more comprehensive and public databases to be open in the future such that inferring novel miRNA-disease associations would achieve a more accurate and stable performance.

## Figures and Tables

**Table 1:** Notations.

Symbol	Description
$N_{m}$	number of miRNAs
$N_{d}$	number of diseases
$N_{l}$	size of latent vector dimension
$R\in {\mathbb{R}}^{N_{m}\times N_{d}}$	miRNA-disease association matrix
$M\in {\mathbb{R}}^{N_{m}\times N_{l}}$	miRNA latent space
$D\in {\mathbb{R}}^{N_{d\;}\times N_{l}}$	disease latent space
$S\in {\mathbb{R}}^{\;N_{m}\times N_{m}}$	miRNA similarity matrix
$W\in {\mathbb{R}}^{\;N_{m}\times N_{m}}$	miRNA expression weight matrix

**Table 2:** Prediction of top 50 colon neoplasms candidates.

Rank	Name	Evidence	Rank	Name	Evidence
1	hsa-let-7a-3	HMDD v2.0	26	hsa-let-7f-2	HMDD v2.0
2	hsa-miR-19a	dbDEMC, HMDD v2.0	27	hsa-miR-205	dbDEMC, HMDD v2.0
3	hsa-let-7f-1	HMDD v2.0	28	hsa-miR-125a	dbDEMC, HMDD v2.0
4	hsa-miR-137	dbDEMC, HMDD v2.0	29	hsa-miR-106a	dbDEMC, HMDD v2.0
5	hsa-let-7a-1	HMDD v2.0	30	hsa-miR-101-1	HMDD v2.0
6	hsa-miR-24-1	HMDD v2.0	31	hsa-miR-365a	HMDD v2.0
7	hsa-miR-141	dbDEMC, HMDD v2.0	32	hsa-miR-21	dbDEMC, HMDD v2.0
8	hsa-miR-30c-2	HMDD v2.0	33	hsa-miR-9-3	HMDD v2.0
9	hsa-miR-128-2	HMDD v2.0	34	hsa-miR-296	dbDEMC, HMDD v2.0
10	hsa-miR-629	HMDD v2.0	35	hsa-miR-493	dbDEMC, HMDD v2.0
11	hsa-miR-486	dbDEMC, HMDD v2.0	36	hsa-miR-142	HMDD v2.0
12	hsa-miR-29b-1	HMDD v2.0	37	hsa-miR-9-2	HMDD v2.0
13	hsa-miR-92a-1	dbDEMC, HMDD v2.0	38	hsa-miR-19b-2	HMDD v2.0
14	hsa-miR-132	dbDEMC, HMDD v2.0	39	hsa-miR-145	dbDEMC, HMDD v2.0
15	hsa-miR-330	HMDD v2.0	40	hsa-miR-218-2	HMDD v2.0
16	hsa-miR-200c	HMDD v2.0	41	hsa-miR-30a	dbDEMC, HMDD v2.0
17	hsa-miR-584	dbDEMC, HMDD v2.0	42	hsa-miR-16-1	dbDEMC, HMDD v2.0
18	hsa-miR-1-1	HMDD v2.0	43	hsa-miR-122	Literature [41]
19	hsa-miR-365b	HMDD v2.0	44	hsa-miR-125b-2	HMDD v2.0
20	hsa-miR-506	dbDEMC, HMDD v2.0	45	hsa-miR-127	dbDEMC, HMDD v2.0
21	hsa-miR-199a	Literature [42]	46	hsa-miR-150	Literature [40]
22	hsa-miR-101-2	HMDD v2.0	47	hsa-miR-502	HMDD v2.0
23	hsa-miR-22	dbDEMC, HMDD v2.0	48	hsa-miR-615	HMDD v2.0
24	hsa-miR-9-1	HMDD v2.0	49	hsa-miR-6815-5p	unconfirmed
25	hsa-miR-155	dbDEMC, HMDD v2.0	50	hsa-miR-16-2	HMDD v2.0

The first and third column correspond to the top 1–25 related miRNAs and 26–50 related miRNAs, respectively.

**Table 3:** Prediction of top 50 kidney neoplasms candidates.

Rank	Name	Evidence	Rank	Name	Evidence
1	hsa-mir-194	dbDEMC	26	hsa-mir-26b	dbDEMC
2	hsa-mir-204	dbDEMC	27	hsa-mir-29b	dbDEMC, miR2Disease
3	hsa-mir-124a	dbDEMC	28	hsa-mir-30e-3p	dbDEMC
4	hsa-mir-199a	dbDEMC, miR2Disease	29	hsa-mir-143	dbDEMC
5	hsa-mir-215	dbDEMC	30	hsa-mir-200a	dbDEMC
6	hsa-mir-210	dbDEMC, miR2Disease	31	hsa-mir-224	dbDEMC
7	hsa-mir-199a*	dbDEMC	32	hsa-mir-30a-3p	dbDEMC
8	hsa-mir-182*	dbDEMC	33	hsa-mir-146a	dbDEMC
9	hsa-mir-30d	dbDEMC	34	hsa-mir-20a	dbDEMC, miR2Disease
10	hsa-mir-15a	dbDEMC, miR2Disease	35	hsa-mir-422a	dbDEMC
11	hsa-mir-136	dbDEMC	36	hsa-mir-130b	dbDEMC
12	hsa-mir-22	dbDEMC	37	hsa-mir-130a	dbDEMC
13	hsa-mir-101	dbDEMC, miR2Disease	38	hsa-mir-455	dbDEMC
14	hsa-mir-320	dbDEMC	39	hsa-mir-489	dbDEMC, miR2Disease
15	hsa-mir-122a	dbDEMC	40	hsa-mir-183	dbDEMC
16	hsa-mir-30c	dbDEMC	41	hsa-mir-30a-5p	dbDEMC
17	hsa-mir-214	dbDEMC, miR2Disease	42	hsa-mir-30b	dbDEMC
18	hsa-mir-198	dbDEMC	43	hsa-mir-139	dbDEMC
19	hsa-mir-107	dbDEMC	44	hsa-mir-181b	dbDEMC
20	hsa-mir-192	dbDEMC	45	hsa-mir-30a	Literature [42]
21	hsa-mir-106a	dbDEMC, miR2Disease	46	hsa-mir-187	dbDEMC
22	hsa-mir-186	dbDEMC	47	hsa-mir-133b	unconfirmed
23	hsa-mir-142	Literature [41]	48	hsa-mir-93	dbDEMC
24	hsa-mir-191	dbDEMC, miR2Disease	49	hsa-let-7e	unconfirmed
25	hsa-mir-422b	dbDEMC	50	hsa-mir-429	dbDEMC

The first and third column correspond to the top 1–25 related miRNAs and 26–50 related miRNAs.

**Table 4:** Prediction of top 50 lymphoma candidates.

Rank	Name	Evidence	Rank	Name	Evidence
1	hsa-miR-138-1	HMDD v2.0	26	hsa-miR-135b	HMDD v2.0, dbDEMC
2	hsa-miR-139	HMDD v2.0, dbDEMC	27	hsa-miR-19b-1	HMDD v2.0
3	hsa-miR-92a-2	HMDD v2.0	28	hsa-miR-101-2	HMDD v2.0
4	hsa-miR-124-1	HMDD v2.0	29	hsa-miR-181a-2	HMDD v2.0
5	hsa-miR-218-2	HMDD v2.0	30	hsa-miR-499a	HMDD v2.0
6	hsa-miR-20b	HMDD v2.0, dbDEMC	31	hsa-miR-122	HMDD v2.0, dbDEMC
7	hsa-miR-29c	HMDD v2.0, dbDEMC	32	hsa-miR-135a-2	HMDD v2.0
8	hsa-miR-16-1	HMDD v2.0	33	hsa-miR-150	HMDD v2.0, dbDEMC
9	hsa-miR-200b	HMDD v2.0, dbDEMC	34	hsa-miR-92a-1	HMDD v2.0
10	hsa-miR-181a-1	HMDD v2.0	35	hsa-miR-550a-2	HMDD v2.0
11	hsa-miR-550a-1	HMDD v2.0	36	hsa-miR-155	HMDD v2.0, dbDEMC
12	hsa-miR-125a	HMDD v2.0, dbDEMC	37	hsa-miR-15a	HMDD v2.0, dbDEMC
13	hsa-miR-24-1	HMDD v2.0	38	hsa-miR-92b	HMDD v2.0, dbDEMC
14	hsa-miR-17	HMDD v2.0, dbDEMC	39	hsa-miR-16-2	HMDD v2.0
15	hsa-miR-133b	HMDD v2.0, dbDEMC	40	hsa-miR-138-2	HMDD v2.0
16	hsa-miR-218-1	HMDD v2.0	41	hsa-miR-18a	HMDD v2.0, dbDEMC
17	hsa-miR-382	unconfirmed	42	hsa-miR-203	HMDD v2.0, dbDEMC
18	hsa-miR-363	HMDD v2.0, dbDEMC	43	hsa-miR-518b	HMDD v2.0, dbDEMC
19	hsa-miR-19b-2	HMDD v2.0	44	hsa-miR-26a-1	HMDD v2.0
20	hsa-miR-146a	HMDD v2.0, dbDEMC	45	hsa-miR-429	unconfirmed
21	hsa-miR-184	HMDD v2.0, dbDEMC	46	hsa-miR-126	HMDD v2.0, dbDEMC
22	hsa-miR-511	unconfirmed	47	hsa-miR-135a-1	HMDD v2.0
23	hsa-miR-101-1	HMDD v2.0	48	hsa-miR-147	unconfirmed
24	hsa-miR-26a-2	HMDD v2.0	49	hsa-miR-210	HMDD v2.0, dbDEMC
25	hsa-miR-21	HMDD v2.0, dbDEMC	50	hsa-mir-320a	unconfirmed

The first and third column correspond to the top 1–25 related miRNAs and 26–50 related miRNAs, respectively.
